# The Impact of Social Media & Technology on Child and Adolescent Mental Health

**Published:** 2025-04-16

**Authors:** Tariq Masri-zada, Suren Martirosyan, Alexander Abdou, Ryan Barbar, Samuel Kades, Hassan Makki, Grant Haley, Devendra K Agrawal

**Affiliations:** 1 Department of Translational Research, College of Osteopathic Medicine of the Pacific, Western University of Health Sciences, Pomona, CA, USA; 2 College of Osteopathic Medicine, William Carey University, Hattiesburg, MS, USA

**Keywords:** Adolescent Mental Health, Attention Deficit Hyperreactivity Disorder, Autism Spectrum Disorder, Body Dysmorphic Disorder, Cognitive-Behavioral Therapy, Cyberbullying, Digital Technology, Dopamine Reward System, Intervention Strategies, Neurodevelopment, Social Media Addiction

## Abstract

The prevalence of mental health disorders among youth and adolescents has been rising at an alarming rate over the past few decades, with conditions such as anxiety, depression, attention deficit hyperreactivity disorder, autism spectrum disorder, and body dysmorphic disorder becoming increasingly common. One contributing factor that has received growing attention is the role of social media and technology in shaping adolescent brain development, behavior, and emotional well-being. While digital platforms provide opportunities for social connection, self-expression, and mental health support, they also introduce significant risks, including compulsive social media use, cyberbullying, unrealistic beauty standards, and exposure to substance-related content. This article explores the complex relationship between digital media use and adolescent mental health, focusing on its neurobiological implications, particularly the role of dopaminergic reward pathways in reinforcing compulsive behaviors. The excessive engagement with digital platforms has been associated with heightened impulsivity, attention deficits, and an increased risk of addiction-like behaviors. Furthermore, the impact of social media on self-esteem and body image has been linked to higher rates of body dysmorphic disorder and a rise in cosmetic procedure considerations, often influenced by digitally altered self-perceptions. The increased portrayal of substance-related content online also raises concerns about the normalization of risky behaviors among impressionable youth. Intervention strategies such as digital detox programs, school-based educational initiatives, parental monitoring, and cognitive-behavioral therapy are crucial in mitigating the adverse effects of excessive social media use. A multidisciplinary approach, integrating policy regulation, digital literacy, and targeted mental health interventions, will be essential in fostering a healthier digital environment for adolescents. Future research should prioritize longitudinal studies to better understand the long-term psychological effects of social media use and to distinguish between adaptive and maladaptive digital behaviors. By addressing these challenges proactively, society can work towards promoting responsible social media engagement in youth while protecting adolescent mental health in this digital age.

## Introduction

The rising prevalence and incidence of mental health disorders such as autism spectrum disorder (ASD), attention deficit hyperactivity disorder (ADHD), anxiety disorders, addiction, and major depressive disorder (MDD) among youth has become a major public health concern. Clinicians and researchers continue to investigate the underlying causes of this trend, with increasing attention on environmental, genetic, and societal factors. One emerging factor that has been speculated to have an impact is the rapid expansion of digital technology and social media. Adolescents today are more connected than ever, spending hours on platforms such as Instagram, TikTok, and Snapchat, which shape their social interactions, self-perception, and neuro-cognitive development. While these digital spaces provide opportunities for connection and self-expression, they also introduce significant challenges, including increased exposure to cyberbullying, unrealistic beauty standards, and compulsive content consumption.

This paper explores the complex relationship between digital media and adolescent mental health, analyzing its impact on disorders such as ASD, ADHD, anxiety, depression, bipolar disorder, and body dysmorphic disorder (BDD). Additionally, it examines the growing concern of social media addiction and its association with substance use behaviors, highlighting both opportunities and challenges in mitigating the negative effects of digital technology on youth well-being.

A study using data from the National Health Interview Survey reported that the prevalence of ASD increased from 0.18% in 1997 to 3.36% in 2019 [1]. Another study found that the prevalence of ASD among individuals aged 3–17 years reached 3.42% in years 2021–2022, continuing a decade-long upward trend [2]. This steady rise in ASD diagnoses could be attributed to multiple factors including broadened diagnostic criteria, improved screening, and better access to care. Advances in screening tools and early intervention programs have played a role in identifying more cases. However, other external influences may have a significant impact, including prenatal exposures, genetic predispositions, and the potential role of digital technology in neurodevelopment. Additionally, the increasing prevalence of ASD places an increasing load on healthcare systems, educational institutions, and social structures to provide adequate support.

Beyond ASD, the addictive nature of social media is emerging as a critical issue, particularly among children and adolescents. Recent studies indicate that approximately 24.4% of adolescents meet the criteria for social media addiction [3]. This compulsive engagement with digital platforms has been associated with increased symptoms of anxiety, depression, and attention disorders, raising concerns about the long-term consequences of excessive screen time. The ease of access and algorithm-driven content personalization contribute to a cycle of prolonged use, reinforcing addictive behaviors similar to those seen in substance use disorders.

Research also suggests that adolescents who spend excessive time on social media (more than three hours per day) are significantly more likely to experience mental health issues, particularly increased symptoms of anxiety and depression [4]. The overuse of platforms like TikTok and Instagram has been linked to lower life satisfaction, compulsive behavior, and an increased risk of developing psychiatric symptoms [5]. While social media and technological advancements like artificial intelligence (AI) provide opportunities for creativity and socialization, these findings indicate that its impact on adolescent mental health is multi-faceted, requiring careful consideration of both its benefits and potential harms.

Additionally, social media exposure is closely linked to adolescent substance use behaviors. Research suggests that adolescents who frequently encounter depictions of alcohol and drug use on these platforms are at higher risk of engaging in these behaviors themselves [6]. This effect is particularly concerning given adolescents’ heightened sensitivity to peer influence and their desire for social acceptance. A systematic review and meta-analysis found that increased social media engagement is associated with a greater likelihood of engaging in health-risk behaviors, including alcohol and drug consumption [3]. The normalization of substance use in digital spaces raises important questions about the role of social media in shaping adolescent decision-making and risk-taking behaviors.

This broad range of findings highlights the need for comprehensive research to better understand the mechanism by which these disorders develop, and this will allow for targeted intervention strategies. Implementing digital literacy programs, promoting healthy online behaviors, and developing targeted interventions are critical steps in mitigating the impact of the digital connection on mental health. As social media continues to evolve, a multi-disciplinary approach by combining education, policy regulation, and mental health support is essential to creating a safer and healthier digital environment for adolescents and youth.

## Neurological Pathways

### Dopaminergic Pathways in Social Media Engagement

The mesolimbic dopaminergic pathway has been associated with addictive behaviors, including drug use and binge eating disorders [7, 8]. This pathway, located in the midbrain’s ventral tegmental area (VTA), has neurons connected between various regions including the amygdala, prefrontal cortex, and nucleus accumbens. Among these, the nucleus accumbens (NAc) is critical to forming reward and aversion behaviors [9]. This dopaminergic circuitry, which evolved to respond to positive and noxious stimuli, may also make individuals, especially the youth, susceptible to compulsive behaviors [10]. The interaction between the brain’s reward system and the modern digital world presents a new dimension for potentially addictive behaviors.

Dopamine plays a key role in the NAc, particularly in the context of instant gratification and social media use, by modulating reward-seeking behavior and impulsive decision-making. According to research, reward-predictive cues, such as notifications on social networking platforms, induce dopamine release in the NAc. This release encourages reward-seeking behavior by increasing cue-evoked excitement in NAc neurons [11]. The prospect of obtaining a reward, such as a like or a comment, causes persistent dopamine release, encouraging the habit of monitoring social media regularly [12].

The growth of social media sites and their use of varied reward schedules, such as likes, comments, and notifications, is consistent with mechanisms that have been proven to influence the mesolimbic system. According to research, the unpredictable nature of these rewards increases dopamine release, boosting the urge for continuing involvement [13]. This type of reward system, like drug use or gambling, perpetuates a loop of obsessive behavior in which the brain seeks the next “hit” of dopamine [13]. Similarly, the consumption of short-form information, which is geared to deliver rapid gratification, exacerbates this trend. Research shows that such rapid reward structures, including short TikTok videos or Instagram reels, can significantly reduce attention control over time [14].

Individuals with pre-existing mental health disorders may have a considerable increase in compulsive behaviors when exposed to digital surroundings. Problematic usage of social networking sites (SNS) has been associated with higher rates of depression, anxiety, stress, obsessive-compulsive disorder (OCD), and attention-deficit/hyperactivity disorder (ADHD) [15]. Furthermore, excessive screen time, particularly with intense and fast-paced content, has been linked to ADHD-related behaviors through dopamine and reward circuit activation [16]. These trends indicate that digital participation can both reflect and exacerbate underlying mental health issues, resulting in a vicious loop that reinforces obsessive behaviors

### The Role of Dopamine in ADHD

ADHD is a neurodevelopmental disorder characterized by persistent patterns of inattention, hyperactivity, and impulsivity that interfere with daily functioning and development and are associated with the dysregulation of the dopamine reinforcement loops. These loops are critically involved in the reinforcement, learning, and behavior modification processes. The current literature proposes an alteration in the tonic and phasic dopamine release signaling system in ADHD patients. Some studies have hypothesized a hyperdopaminergic state in ADHD patients, while others have proposed a hypodopaminergic state to explain the pathophysiology associated with the condition.

Badgaiyan et al. most recently demonstrated that ADHD is associated with both the attenuated tonic dopamine release and enhanced phasic dopamine release in the right caudate nucleus [17]. This dysregulation leads to ADHD patients having difficulty in executing reinforcement learning, particularly when it comes to error processing and reward anticipation. It has also been demonstrated by Chevrier et al. that patients with ADHD demonstrate disruptions to the normal reinforcement learning process, especially during the post-error slowing, with altered activity in the ventral and dorsal striatum as well as a heightened amygdala response to errors [18]. Post-error slowing in ADHD refers to the reduced tendency of individuals with ADHD to slow down their responses after they have made a mistake, compared to people without ADHD, which indicates a potential impairment in their ability to recognize and adapt to errors and regulate their behavior.

There have been computational studies performed that also show low striatal dopamine levels in ADHD patients which leads to deficits in goal-oriented learning with positive reinforcements, which is often enhanced exogenously with stimulant medications [19]. This is further validated by the findings that stimulant medications can improve task performance and reward-learning rates in ADHD patients by specifically modulating dopaminergic activity in the brain [20]. By further investigating the complex interplay of dopamine in the disease, there could be alternate targets for drugs developed to help individuals manage this disease.

Dopamine dysregulation has a major impact on ADHD symptoms in digital environments, altering brain areas involved in impulse control, decision-making, and reward processing [21]. This dysregulation makes it difficult for those with ADHD to resist distractions and delay satisfaction, which is especially troublesome in digital settings [22]. These environments are intended to provide rapid rewards, promoting impulsive behavior and making it more difficult to concentrate on tasks that demand sustained attention [14].

### Serotonin and Emotional Dysregulation

Aside from disrupting attention control, extended exposure to these digital reward systems may have larger implications for motivation and self-esteem. Placing significant value on input from others on social media sites like Instagram, such as likes, followers, and comments, has been associated with reduced self-esteem and a reduced appraisal of social status [23]. This external validation-seeking behavior exacerbates the cycle of compulsive social media involvement, as people are constantly seeking confirmation. The addictive nature of smartphone use exacerbates this loop, with excessive reassurance-seeking behavior (ERSB) mediating the link between rumination and problematic use of these platforms [24].

Serotonin is a key neurotransmitter in the regulation of the external reward pathway and has a critical neurodevelopmental impact on youth. Serotonin acts as a growth factor during early development and genetic variations of the signaling pathway have been linked to individual susceptibility to psychiatric disorders [25]. Particularly, the interaction between serotonin and dopamine has been studied and linked to the addiction pathway having an impact on impulsivity. This can predispose adolescents to drug experimentation along with subsequent dependency in response to anxiety and depression [26].

Disruptions in serotonergic signaling can contribute to emotional dysregulation and increased susceptibility to external influences, such as social media and online interactions. Social media use has been linked to increased depression and lower self-esteem, particularly in adolescents with low emotional self-efficacy [27]. Given serotonin’s role in stress resilience and emotional regulation, deficits in this neurotransmitter may exacerbate the negative effects of online interactions, leading to increased vulnerability to negative feedback [27]. Additionally, online social evaluation significantly impacts mood and cognition in young individuals, reinforcing the notion that serotonin-controlled emotional responses can be dysregulated by digital social interactions [28]. Understanding these mechanisms is essential for developing interventions that strengthen emotional resilience and mitigate the negative effects of excessive social media use and addiction in youth.

In addition, serotonin imbalances, commonly observed in depression and anxiety, are exacerbated by online interactions [29]. These imbalances exacerbate mental health issues among teenagers, particularly in those who are prone to such illnesses [30, 15]. As demonstrated by Lee et al. (2008), short allelic variants of the serotonin transporter gene may be associated with internet addiction. This shows that those with serotonin dysregulation are more prone to internet-related problems, such as social media addiction [31]. When combined with past research, it is obvious that this loop of social media use caused by serotonin dysregulation might contribute to and perpetuate depression symptoms.

### Substance Addiction and Social Media Habits

The neurobiological similarities between substance addiction and compulsive social media usage are striking, particularly in terms of prefrontal cortex (PFC) dysfunction, which is critical for impulse control and decision-making [32, 33]. According to Von Deenen et al. (2022), excessive internet gaming and nicotine addiction in adolescents result in similar deficits in functional connectivity throughout the frontostriatal network, which is required for both cognitive regulation and reward processing [34]. These findings indicate that similar disturbances can also affect social media use, where impulsivity and reward-seeking behaviors are important to participation, as well as other substances of abuse, exhibiting clear neurological connections.

The hypothalamic-pituitary-adrenal (HPA) axis, which regulates the stress response of the body, is another important system impacted by addiction-like behaviors such as excessive social media use. Chronic activation of the HPA axis causes sustained cortisol to increase and altered stress responses, which is a common occurrence in substance addiction [35]. This chronic stress reaction can worsen obsessive behaviors and render people more susceptible to addictive habits. Excessive screen time, particularly on social media, has been found to cause chronic activation of the HPA axis, which mirrors the stress dysregulation seen in substance use [36]. Additionally, using social media following a stressful incident can hinder cortisol recovery, exacerbating stress dysregulation and perpetuating the dysfunctional cycle [37]. The prolonged activation of the HPA axis in these contexts can impair cognitive function and exacerbate the cycle of addiction by reinforcing reward-seeking behaviors and impulsivity.

## Thematic Findings from Literature

### Depression, Anxiety, and Social Comparison in a Digital Age

The profound influence of social media on adolescent mental health, particularly its relationship with depression and anxiety, underscores the urgent need for further research in this area. Excessive social media use has been associated with increased loneliness, social comparison, and fear of missing out (FOMO), all of which are risk factors for developing or exacerbating depression and anxiety in adolescents [38]. Studies indicate that adolescents who spend significant time on social media are more likely to experience negative mental health outcomes, including decreased self-esteem and increased feelings of loneliness or social isolation [39]. Problematic or addictive patterns of social networking have also been documented and linked to psychological distress in adolescents [40].

A central factor contributing to these negative effects is social comparison. Social media platforms curate idealized portrayals of reality, often leading users to compare their lives unfavorably with others. This constant exposure to thoughtfully selected content has been linked to increased symptoms of depression, particularly among female adolescents, who may be more vulnerable to body image concerns [41]. Social media’s reinforcement of unattainable beauty standards can contribute to heightened anxiety, disordered eating behaviors, and depressive symptoms in teenage girls [42]. Moreover, frequent engagement with appearance-focused content can exacerbate self-consciousness and social withdrawal, worsening anxiety in social situations [43].

While social comparison affects both genders, adolescent males may experience distress related to perceived success, social status, or physical fitness. The pressure to conform to hyper-masculine ideals, such as extreme muscularity, financial success, or dominance, can contribute to body dissatisfaction and self-esteem issues, sometimes leading to conditions like muscle dysmorphia [42]. Societal expectations often pressure young males to maintain emotional stoicism, discouraging them from seeking support. This reluctance can lead to increased social withdrawal and contribute to depressive symptoms [43]. Meanwhile, female adolescents may experience heightened stress from online bullying, cyberstalking, or social exclusion, all of which are linked to increased anxiety and depressive symptoms [44].

Another growing concern is how social media shapes adolescent self-esteem, particularly through online validation. Many teens, both boys and girls, tie their self-worth to the number of likes, comments, and followers they receive [45]. When their posts do not get enough engagement, or they feel ignored online, it can lead to feelings of anxiety, rejection, and self-doubt. The pressure to present a ‘perfect’ version of themselves online only adds to the stress. Social media can make teenagers feel like they must always look their best, say the right things, and keep up with trends. This constant need for approval can take a toll on their mental health, creating a cycle of anxiety and self-doubt.

Beyond issues of self-esteem, social media is also changing how teens communicate in everyday life. Many adolescents now feel more comfortable texting or messaging on social platforms than engaging in face-to-face conversations. As a result, in-person interactions can feel awkward or even anxiety-inducing [41]. Over time, heavy reliance on digital communication can make it harder to build meaningful relationships and navigate offline social situations. For teens already experiencing social anxiety, the ability to curate and control online interactions may reinforce avoidance behaviors, leading to greater isolation and increased stress during in-person exchanges [46]. Social media can also create a false sense of connection—making teens feel socially engaged while they continue to struggle with genuine connection offline. Studies show that adolescents who spend excessive time on social media often feel more isolated despite being constantly connected online [39].

When teens spend less time talking face-to-face, they miss key opportunities to build social skills. When most of their communication happens through screens, they do not get as much practice handling disagreements, expressing emotions, or navigating in-person conversations. Over time, this can make real-world interactions feel even more stressful, especially for those who already struggle with social anxiety. Without these everyday social experiences, it becomes harder to build confidence, manage stress, and develop the coping skills needed for healthy relationships, which can make symptoms of social anxiety and generalized anxiety disorder even worse [44].

While these risks are significant, it is crucial to recognize that social media is not inherently harmful. Its impact largely depends on how it is used, and in some cases, it can serve as a valuable resource for mental health support. Online mental health communities provide peer encouragement, psychoeducation, and accessible coping strategies, offering support for individuals struggling with anxiety and depression [46]. Furthermore, platforms designed to foster positive interactions and community engagement have been found to reduce loneliness and promote social connectedness, mitigating some of the negative effects of excessive social media use [44]. With continued efforts in content moderation, algorithmic design, and mental health advocacy, there is hope for creating safer online spaces for adolescents.

### Autism Spectrum Disorder in the Digital Age

Online communication provides an accessible way to engage socially for individuals with ASD who normally have adversity with the sensory challenges of face-to-face interactions. Digital platforms remove barriers such as loud environments, unexpected physical contact, and rapid conversational shifts, allowing autistic individuals to interact at their own pace and in a controlled setting [47]. Social media, messaging apps, and online forums can serve as safe spaces where autistic individuals feel more comfortable expressing themselves without the immediate pressures of in-person social norms.

Despite the benefits of online communication, autistic individuals may face challenges in interpreting digital social cues. The absence of facial expressions, body language, and vocal tone can make it difficult to discern sarcasm, subtext, or emotional intent conveyed through text [48]. Additionally, emojis, which are often used to convey emotion or humor, may be misinterpreted or misunderstood. This can lead to difficulties in online conversations, mirroring the social challenges many individuals with ASD experience in real-life interactions.

Autistic individuals often develop intense interests in specific topics, and the accessibility of digital content can facilitate deep engagement with these interests. While this can be a positive outlet for learning and connection, it also presents a risk of excessive screen time and potential digital addiction. Studies have suggested that children with ASD are more prone to prolonged screen exposure, which may contribute to difficulties in socialization and behavioral regulation [49]. The immersive nature of digital platforms, particularly gaming and social media, may reinforce hyperfixation tendencies, making it challenging for individuals to disengage from screens.

The relationship between screen time and Autism Spectrum Disorder (ASD) has been a topic of growing research interest. Several studies suggest that increased screen exposure in early childhood may be associated with a higher likelihood of ASD diagnosis or exacerbation of symptoms in children already diagnosed with ASD. However, the nature of this correlation remains complex, with debates surrounding causation, bidirectional influence, and confounding variables.

Multiple studies have explored whether prolonged screen exposure contributes to the development of ASD-related traits. A large-scale study from the Japan Environment and Children’s Study [50] found that children exposed to excessive screen time at one year of age had an increased risk of ASD diagnoses by age three. This aligns with other research suggesting that early and excessive digital exposure may interfere with key developmental processes, particularly in brain regions responsible for social interaction, language acquisition, and executive function. Additionally, Yuan et al. (2024) conducted a comprehensive systematic review, highlighting that increased screen time is associated with altered neural development, particularly in areas governing social cognition. Findings suggest that excessive digital engagement during critical neurodevelopmental periods may lead to deficits in social engagement and communication skills, which are core features of ASD [51].

Neuroimaging studies provide further evidence that prolonged screen exposure may influence brain connectivity patterns in autistic individuals. Xue et al. (2024) found that preschool children with ASD who had high screen exposure exhibited altered intra- and inter-network functional connectivity in brain regions associated with attention, social interaction, and emotional regulation [52]. These findings suggest that excessive screen time may disrupt neural pathways essential for typical social and cognitive development.

Similarly, Dong et al. (2021) examined the correlation between screen time and developmental quotients (DQs) in children with ASD. The study found that higher screen exposure was linked to lower DQs, suggesting potential delays in cognitive, language, and motor development [49]. The mechanism behind this correlation is thought to involve the passive nature of screen interactions, which may replace real-world social experiences critical for neurodevelopment.

While studies suggest a link between increased screen time and ASD, it is important to consider that the relationship may be bidirectional rather than purely causal. Children predisposed to ASD may naturally gravitate toward screens due to a preference for predictability, as digital environments offer structured, repetitive, and predictable interactions that can be comforting for individuals with ASD. Additionally, screens provide an alternative to overwhelming sensory inputs from real-world social settings, helping autistic individuals avoid excessive sensory stimulation. Many autistic individuals also exhibit hyperfixation on specific topics, and digital platforms offer endless access to related content, reinforcing prolonged screen engagement. Ophir et al. (2023) noted that while screen exposure may exacerbate social withdrawal and communication difficulties, it remains unclear whether increased screen time contributes to ASD onset or if children with ASD are more inclined toward screen-based activities [53].

The growing body of research suggests that excessive screen time, particularly in early childhood, may have implications for ASD symptoms and neurodevelopment, but it is crucial to approach these findings with careful analysis. Factors such as genetic predisposition, parental involvement, and quality of screen content all play a role in shaping developmental outcomes. To mitigate potential risks, experts recommend balancing screen time with real-world interactions by encouraging face-to-face communication and physical activities that support social and cognitive development. Parental mediation is also essential in monitoring and guiding screen use to ensure engagement with educational and interactive content rather than passive consumption. Furthermore, early intervention plays a key role in identifying and addressing ASD symptoms at an early stage to provide targeted support, minimizing reliance on digital engagement as a coping mechanism.

While research continues to investigate the correlation between increased screen time and ASD, current findings suggest that excessive digital exposure may influence social development, brain synaptic connections, and ASD symptomatology. However, given the likelihood of bidirectional influence, further studies are needed to clarify whether screen time plays a causal role in ASD development or if autistic individuals are naturally more inclined toward screen-based activities. Patients undergoing ASD may have an increased risk of screen time consumption due to their addictive personalities which predisposes patients to an increased chance of ASD due to developmental issues. We also predispose that patients with ASD may have an increased incidence of other developmental disorders such as body dysmorphia and other conditions including dyslexia, autonomic nervous systemic dysfunction, and bipolar disorder.

### Bipolar Disorder in a Digital Age

The relationship between social media and bipolar disorder is complex, with the potential for both beneficial and harmful effects. Often, individuals experiencing mania engage in impulsive online behaviors, such as oversharing personal information, excessive posting, and compulsive online spending, which can have lasting consequences on their reputation, financial stability, and personal relationships [54]. These impulsive behaviors frequently lead to regret and distress once the individual transitions into a depressive phase, exacerbating feelings of shame and social withdrawal [55].

Additionally, emotionally charged content on social media—such as distressing news or negative interactions—may act as a trigger for mood dysregulation, particularly in individuals vulnerable to mental health conditions like bipolar disorder. Such digital exposures have been linked to changes in emotional states and may exacerbate manic or depressive episodes [56]. Algorithms designed to maximize engagement may contribute to excessive screen time, disrupting circadian rhythms and reducing sleep quality. Since sleep disturbances are a well-established trigger for both manic and depressive episodes, prolonged nighttime social media use can significantly impact mood stability [57].

While these risks highlight the challenges social media poses for individuals with bipolar disorder, digital mood-tracking applications have emerged as a promising tool for self-monitoring in bipolar disorder that can support mental health and treatment adherence. These apps allow individuals to track mood fluctuations, medication adherence, and sleep patterns, providing insights into treatment strategies [58]. Some studies suggest that when used in conjunction with clinical interventions, digital tools such as smartphone-based monitoring can enhance symptom tracking and may help reduce relapse rates in individuals with bipolar disorder [59].

### Increased Screen Time and its Impact on the Attention Span in ADHD Patients

In the United States, the prevalence of diagnosed ADHD among children and adolescents increased from 6.1% in 1997–1998 to 10.5% in 2022, which proved to be significantly observed across all subgroups by age, sex, race/ethnicity, family income, and geographic regions (60, 61). Several studies have shown that recent changes in screen time have been associated with the exacerbation of ADHD symptoms. Wallace et al. showed that increasing the screen time in adolescents with ADHD was associated with a significant increase in impulsivity and to a lesser extent an increase in their inattention and hyperactivity [62]. Abdoli et. al showed that during the COVID-19 pandemic when screen time was also increased, patients with ADHD had a heightened impact on their inattention and hyperactivity compared to the individual without the disease which further validated the negative impact of prolonged screen time. The effects of screen time were also studied across a four-decade span and have found a small but significant relationship between screen media use and ADHD behaviors, further highlighting the importance of monitoring and managing screen time in this population to improve their attention span [63].

Additionally, the studies were conducted on screen time, specifically in the setting of social media. Wallace et al. found that increased social media was associated with ADHD symptom exacerbation, and patients were more likely to be impulsive as well as have response inhibition deficits [62]. Özkan et al further studied social media effects on ADHD patients with a focus on individuals who exhibited problematic social media use [64]. These individuals were excessively and compulsively engaged on social media platforms and exhibited addiction-like symptoms, such as withdrawal, tolerance, negative impacts on mental health, and impairment of overall well-being in the setting of work and personal relationships. These individuals exhibited the highest level of ADHD symptoms in adolescents. Boer at al, further investigated problematic social media use and found that this was linked to increased ADHD symptoms over time, more than in patients who used social media platforms with long sessions and frequent episodes, referred to as intense social media use [65].

There are some online productivity tools available for patients with ADHD such as the EndeavorRX tool, which was the first FDA-approved game-based digital therapeutic for pediatric populations, that was designed to improve the attention in patients with gameplay [66, 67]. Gamified attention training incorporates the game elements into cognitive training tasks which can help enhance engagement and motivation. These programs have built-in algorithms to adjust the level of difficulty of the task so that it remains challenging yet achievable. For instance, with EndeavorRX, the software can generate conflict at updated difficulty levels during a multitasking mini-game. This has been shown to improve attention and reduce ADHD symptoms. Similar platforms are being designed to be engaging, fun, adaptive, and overall therapeutic for ADHD patients.

### Body Dysmorphic Disorder and Photographic Filters

Body Dysmorphic Disorder is a mental health condition in which an individual has an obsessive precaution with the flaws they can perceive in their physical appearance, which usually unnoticed by others or are considered minor. This focus can generally lead to distress in the patient which interferes with their social, occupational, or academic endeavors. In the social media age over the last decade, the hyperfocus on staying up to date, connected, and sharing only the best personal images of one’s self has led people to question whether or not this has created an unprecedented increase in body dysmorphic disorders.

The concept of “Snapchat dysmorphia” refers to the phenomenon in which individuals on the platform seek cosmetic procedures in hopes that they can better resemble the filtered selfies being generated on the application. This trend has increased over the years with social media advancement and the widespread access to photo-editing apps on cellular devices which can encourage one to distort their natural appearance, leading to the ensuing dissatisfaction when those standards are not met in reality [68, 69, 70].

Further studies have shown that social media platforms like Snapchat, Instagram, and Facebook have influenced individuals’ decisions to undergo cosmetic procedures. Seetan et al. showed that individuals using these platforms more frequently were more inclined to pursue aesthetic treatments, with Snapchat being the most notable influencer [71]. In addition, Rahman et al. showed that the more engaged the individual was on a social media platform, the more likely they were to consider enhancement procedures, suggesting that spending increased time looking at idealized images could be one of the factors advancing this process [72].

With the advancement of artificial intelligence (AI), camera filtering can now enhance and modify facial features, which can create an unrealistic beauty standard and increase the expectations of users to meet that standard. This phenomenon is presented and highlighted by researchers like Taritsa et al, who found that AI-enhanced images significantly raised expectations for plastic surgery outcomes, leading to lower satisfaction after surgical procedures that do not meet unrealistic expectations [73]. In addition, Hermans et al. showed that individuals more engaged with filtering and editing their pictures were more concerned with cosmetic enhancements to achieve the filtered appearance in real life, especially among young adult populations [74]. Furthermore, the increased use of filters, found to be especially the case during the COVID-19 pandemic, correlated with worsening self-perception and anxiety, particularly in young individuals, according to Silence et al.

The increasing incidence of selfie-taking has also been problematic to the body dysmorphia disorder prevalence. Studies have shown that selfie-taking as well as viewing of photography is linked to increased body dissatisfaction and consideration of cosmetic repairs. Li et al. found that selfie behaviors, especially viewing one’s selfies, is a key factor affecting the consideration of surgical procedures among teenage girls, due to appearance comparisons and body dissatisfaction [75]. Additionally, Lyu et al. proved that selfie behavior in the adolescent population predicted higher levels of cosmetic surgery consideration, which was mediated by the physical appearance comparisons to the individuals they looked up to [76].

### Cyberbullying and Adolescent Mental Health

Cyberbullying has increased significantly in recent years, emerging as a major contributor to deteriorating mental health in adolescents. The widespread use of social media platforms has facilitated anonymity-driven harassment, leading to severe psychological distress, including depression, anxiety, and self-harm among young individuals.

The rise of online anonymity has facilitated an increase in cyberbullying, which has been directly linked to self-harm behaviors among adolescents [77]. Anonymity allows perpetrators to evade accountability, allowing them to engage in more aggressive and harmful behaviors [78]. Victims of cyberbullying are at a significantly higher risk of self-harm and suicidal ideation, particularly when exposure is chronic and severe [79]. Research suggests that adolescents who experience cyberbullying often feel powerless, as the digital nature of harassment makes it difficult to escape [80]. Furthermore, social media platforms’ failure to adequately address harmful content exacerbates this crisis, as harmful messages and images can be rapidly disseminated and repeatedly viewed [81]. This cycle of exposure increases distress and negatively impacts mental health.

Negative peer interactions, particularly in the form of cyberbullying, can have profound effects on adolescent self-esteem and overall psychological well-being. Victims often experience social isolation, anxiety, and depression, which contribute to a decline in self-worth and personal confidence [82]. The persistent and public nature of online harassment differentiates it from traditional bullying, as the audience is potentially unlimited, leading to increased humiliation and long-lasting emotional scars [83]. Studies show that adolescents involved in cyberbullying, whether as victims or perpetrators, report lower life satisfaction and higher levels of distress [84].

Additionally, the visibility of idealized online personas on social media contributes to self-comparison and unrealistic self-expectations, further compounding feelings of inadequacy [85]. The psychological distress from cyberbullying can also manifest in reduced academic performance and decreased participation in offline social activities, reinforcing a cycle of isolation and emotional suffering [86].

Despite increased awareness, moderating harmful content on social media platforms remains a critical challenge. Automated moderation systems often struggle to differentiate between harmful content and casual interactions, leading to inconsistencies in enforcement [81]. Research highlights that current content moderation practices lack efficiency in identifying evolving forms of cyberbullying, such as subtle harassment, exclusion, and coded language used to target individuals [80]. Moreover, victims of cyberbullying frequently report dissatisfaction with the response from platform administrators, as harmful content often remains visible even after being reported [86]. Studies have also found that social media policies vary significantly across platforms, leading to gaps in protection for at-risk users [85]. The lack of effective moderation allows cyberbullying to persist unchecked, leaving many adolescents vulnerable to its damaging effects [84].

Additionally, research suggests that exposure to cyberbullying can lead to long-term psychological distress, increasing susceptibility to depression and other mental health disorders [78, 79]. More comprehensive and proactive moderation strategies, including AI-driven detection, improved reporting systems, and stricter regulations, are necessary to mitigate harmful impact of cyberbullying [82, 77].

Cyberbullying presents a significant challenge to adolescent mental health, contributing to self-harm risks, diminished self-esteem, and social withdrawal. The anonymity provided by digital platforms enables harassment to thrive, while inadequate content moderation allows harmful behaviors to persist. Addressing these challenges requires a multi-pronged approach that includes improved regulatory policies, better AI-driven content moderation, increased parental and educator involvement, and enhanced mental health support for victims. Future research should focus on developing more effective intervention strategies to protect adolescents in online spaces while promoting digital literacy and resilience-building measures

## Treatment Strategies, Behavioral Modifications, and Reducing Negative Impact of Social Media

### Behavioral and Psychosocial Interventions

#### Cognitive-Behavioral Therapy for Internet Addiction

In the context of internet addiction, Young developed the Cognitive-Behavioral Therapy for Internet Addiction (CBT-IA) model, which uniquely combines CBT with harm reduction therapy (HRT). As validated in a study of 128 clients, CBT-IA includes behavior modification to control compulsive Internet use, cognitive restructuring to address denial and rationalizations, and harm reduction techniques to treat co-morbid issues associated with the disorder. The study demonstrated that over 95% of clients were able to manage symptoms at the end of twelve weekly sessions, and 78% sustained recovery six months following treatment [87]. This tailored approach addresses the unique challenges of internet addiction, where complete abstinence is not an typically option in a digital world.

For anxiety disorders, Carpenter et al. (2018) conducted a meta-analysis of randomized placebo-controlled trials and found that CBT consistently reduced symptoms across various anxiety-related conditions. The study highlighted CBT’s ability to help individuals challenge cognitive distortions and develop coping strategies, leading to improved outcomes [88]. Similarly, Bandelow et al. (2020) examined long-term outcomes and found that CBT’s benefits persisted over time, particularly for conditions like social anxiety disorder and post-traumatic stress disorder [89]. These findings present CBT’s efficacy as a structured and adaptable treatment for anxiety.

Regarding self-esteem issues, Kolubinski et al. (2018) conducted a systematic review and meta-analysis of CBT-based interventions based on the Fennell model of low self-esteem. The study found that CBT was effective in addressing negative self-beliefs and maladaptive thought patterns, leading to improvements in self-esteem [90]. The structured nature of CBT allows individuals to identify and modify these patterns, fostering a healthier self-concept.

CBT demonstrates significant efficacy in treating compulsive internet use, anxiety disorders, and self-esteem issues. Its structured, evidence-based approach makes it a versatile treatment option for these psychological challenges. However, the effectiveness of CBT varies across conditions, and future research should focus on developing more targeted interventions, improving treatment adherence, and evaluating long-term outcomes to enhance its application across diverse populations.

#### Behavioral Interventions for People with ASD

ASD is a complex neurodevelopmental condition characterized by social communication deficits and repetitive behaviors. Treatment for ASD involves both behavioral interventions and pharmacological approaches, with medication primarily targeting co-occurring symptoms rather than core ASD traits. The most effective management strategies integrate multiple treatment modalities, tailoring interventions to the individual’s specific needs.

Behavioral interventions remain foundational to ASD treatment, with approaches such as Applied Behavior Analysis (ABA), Early Start Denver Model (ESDM), and Pivotal Response Training (PRT) demonstrating efficacy in improving communication, social skills, and adaptive behaviors [91]. Intensive early interventions, particularly those initiated before the age of five, show the greatest benefits in language development and cognitive functioning. These therapies are often individualized to address the child’s unique strengths and deficits.

Social Skills Training (SST) has been established as an effective intervention for individuals with ASD, addressing challenges in social interaction and communication. Research consistently supports the efficacy of SST in improving social competence and relationships for individuals with ASD, with meta-analyses reporting medium to high effect sizes for these interventions. For instance, a meta-analysis comparing face-to-face SST (F2F-SST) and technology-based interventions (BITs-SST) found no significant differences in effectiveness between the two approaches, with effect sizes consistently in the medium to high range [92].

SST employs structured techniques such as role-playing, modeling, social stories, and visual supports to teach and reinforce social skills. These methods provide opportunities for individuals with ASD to practice social behaviors in a controlled environment, helping them generalize these skills across various settings. While both F2F-SST and BITs-SST are effective, each presents unique challenges. For example, telehealth-based SST requires greater preparation time and effort from parents compared to F2F sessions [93].

The intensity and duration of SST programs significantly impact outcomes. More intensive interventions, particularly those exceeding 50 hours, have been shown to produce greater gains in social skills. This highlights the importance of sustained engagement to achieve meaningful improvements. However, specific details about the impact of duration on outcomes are not provided in the available sources.

#### Digital Detox Interventions

The COVID-19 pandemic has significantly impacted screen time usage and digital habits, particularly among families with young children. Recent studies have explored the effects of digital detox interventions, parental strategies for managing screen time, and the challenges of navigating technology use during this unprecedented period.

A systematic review and meta-analysis conducted in 2024 examined the impacts of digital social media detox on mental health outcomes. The study found a significant reduction in depressive symptoms following the gradual reduction in digital or social media usage. However, the effects on other mental health indicators such as overall well-being, life satisfaction, and stress were not statistically significant [94].

Parental use of routines and limit-setting has been associated with children’s screen time during the pandemic. A longitudinal study of Canadian children under 12 years of age from May 2020 to May 2021 revealed that lower parental use of routines and setting limits was associated with higher child screen time and lower odds of meeting the Canadian 24-hour screen time guideline, particularly among children aged 5 years and older [95].

Digital detox interventions have been proposed as a potential solution to mitigate the negative impacts of smartphone use on well-being and social relationships. A systematic literature review of 21 studies with 3,625 participants, found mixed results, with some studies reporting positive intervention effects and others showing no effect or even negative consequences for well-being (96). Practical strategies for managing screen time after periods of increased usage, such as summer vacations, include setting clear rules, creating tech-free zones, and involving children in the process of establishing boundaries. Explaining the reasoning behind screen time limits can increase the youth’s compliance with these rules [97].

Research on digital detox and mindfulness practices suggests that strategies such as mindfulness and gratitude may be effective in promoting healthier digital habits [98]. However, parents of young children have reported challenges in navigating screen time during the pandemic, highlighting the need for balanced approaches that consider the realities of modern family life [99].

#### Mitigating Harmful Effects of Social Media

Content moderation and cyberbullying prevention have become critical challenges in the digital age, with platforms increasingly turning to artificial intelligence (AI) to manage vast amounts of user-generated content. Recent advancements in AI have revolutionized content moderation practices, offering scalable solutions to detect and filter harmful content in real-time.

AI-powered content moderation systems utilize machine learning algorithms, natural language processing, and computer vision to automatically analyze and classify potentially harmful content [99]. These systems can process enormous volumes of data, significantly outpacing human moderators in speed and efficiency. Major social media platforms have implemented AI-driven safety measures to combat cyberbullying and harmful content. For instance, Facebook employs in-house AI systems such as Deep Text, FastTex, multi-lingual model XLM-R (RoBERTa), and Reasoning Intention-Oriented Objects (RIO) to detect and filter unwanted content [100].

While AI-driven moderation offers benefits such as scalability, consistency, and reduced exposure to harmful content for human moderators, it also presents challenges. These include potential algorithmic bias, issues with accuracy in detecting nuanced content, and concerns about transparency and accountability [101]. To address these challenges, many platforms are adopting a hybrid approach that combines AI-driven moderation with human oversight [102].

Recent studies have shown that anti-cyberbullying programs can reduce cyberbullying perpetration by approximately 10–15% and cyberbullying victimization by approximately 14% [103]. However, the effectiveness of these interventions varies, and more research is needed to develop targeted, evidence-based approaches.

As the field of AI-driven content moderation continues to evolve, researchers and tech companies are exploring more advanced solutions. For example, some startups are developing AI models trained with diverse perspectives to better understand and moderate content related to marginalized communities. Additionally, voice and audio moderation tools are being developed to analyze real-time communication and identify harassment in live audio interactions [104].

While AI-driven content moderation and reporting systems have significantly improved platforms’ ability to limit cyberbullying and harmful content, challenges remain. Future developments in this field must focus on improving the accuracy and fairness of AI models, enhancing transparency, and balancing between automated and human moderation to create safer online environments [102].

#### School-based Programs and Social-emotional Learning

Educational and school-based programs have emerged as crucial interventions in addressing cyberbullying and promoting digital literacy among students. These programs often incorporate social-emotional learning (SEL) initiatives, which have shown promise in reducing bullying behaviors and fostering a positive school climate.

Recent studies have demonstrated the effectiveness of school-based cyberbullying prevention programs. A systematic review and meta-analysis found that antibullying programs are effective in reducing bullying perpetration by approximately 18–19% and bullying victimization by 15–16% [105]. These findings highlight the potential impact of well-designed interventions in school settings.

Social-emotional learning has been identified as a key component in successful bullying prevention efforts. SEL programs typically focus on developing five main competencies: self-awareness, self-management, social awareness, responsible decision-making, and relationship skills [106]. These skills are crucial in helping young school-age students navigate online interactions and respond appropriately to potential cyberbullying situations.

Research has shown that SEL interventions can have significant effects on students’ behavior and attitudes. A study evaluating the STAnD anti-cyberbullying program found that participants demonstrated an increased willingness to seek help and defend others after the intervention, with larger changes observed among lower primary school students [107]. This suggests that early intervention may be particularly effective in shaping positive online behaviors.

The integration of SEL into school-wide approaches has also shown promise. A large-scale survey study published in School Psychology examined how school climate and SEL competencies influenced adolescents’ cyberbullying experiences. The study found that specific SEL competencies, such as self-management and responsible decision-making, were associated with reduced cyberbullying victimization [108]. These findings highlight the importance of tailoring SEL interventions to focus on specific competencies and considering factors such as gender and grade level.

While the effectiveness of these programs is evident, challenges remain in their implementation and long-term impact. A study evaluating a European-funded preventive program (TABBY) found that while students’ cyberbullying engagement decreased significantly immediately after and six months following the intervention, the effects were more pronounced for students with special educational needs [109]. This highlights the need for tailored approaches that consider different types of students.

Educational and school-based programs that incorporate digital literacy and social-emotional learning initiatives have shown significant potential in preventing cyberbullying and promoting positive online behaviors. Future research should focus on developing comprehensive, tailored interventions that address the evolving nature of online interactions and consider the diverse needs of student populations.

#### Parental Control

Parental and peer support strategies play a crucial role in preventing and addressing cyberbullying among youth. Recent research has highlighted the importance of open communication, parental limitations, and peer-led mental health advocacy in fostering a safer online environment for children and adolescents.

Open and supportive communication between parents and children is critical in reducing cyberbullying and online aggression. Engaging in active conversations about digital experiences helps shape positive social norms, preventing cyber aggression in adolescents. Actively discussing online interactions fosters trust, encouraging children to report cyberbullying and seek parental support. Additionally, when parents understand digital platforms, they can provide informed guidance, promoting safer online behaviors [110].

Parental monitoring and control strategies are recognized as essential tools for guiding children’s online experiences. Research highlights that setting clear boundaries and maintaining open communication increases the effectiveness of these measures. Parents can implement strategies such as restricting access to certain websites, monitoring online activity, and setting time limits for device usage. However, building trust through honest discussions about online safety is critical. Excessive monitoring without open communication can weaken the parent-child relationship and reduce children’s willingness to disclose online concerns, highlighting the need for a balanced approach [110].

Peer-led mental health advocacy has emerged as a promising approach to mitigate the harmful effects of social media on mental health. A systematic review published in the Journal of Medical Internet Research Mental Health highlighted the potential of digital peer support systems in promoting mental well-being [111]. The review emphasized the importance of designing these systems to facilitate meaningful connections and support among users.

In conclusion, effective strategies for preventing and addressing cyberbullying involve a combination of parental support, peer-led initiatives, and educational programs. Open communication, proper use of parental controls, peer advocacy, and social-emotional learning all contribute to creating safer online environments for youth. Future research should focus on evaluating the long-term effectiveness of these strategies and developing integrated approaches that leverage both parental and peer support systems.

## Medical and Pharmacological Treatments

### Pharmacological Treatments for ADHD

Attention-Deficit/Hyperactivity Disorder (ADHD) is a common neurodevelopmental disorder affecting children and adolescents, with treatment approaches including both pharmacological and behavioral interventions. Medications, particularly stimulants, remain the primary option for managing symptoms, while non-stimulant alternatives provide additional choices for those who cannot tolerate stimulants. Combining medication with behavioral therapies has been shown to improve overall functioning and long-term outcomes.

Stimulant medications, such as methylphenidate and amphetamines, are the most frequently prescribed treatments for ADHD, targeting the central nervous system to improve attention, impulse control, and hyperactivity. Studies have consistently demonstrated that stimulant medications are more effective than behavioral therapy or cognitive training alone in reducing symptoms [112]. Extended-release formulations offer sustained symptom control, making them particularly beneficial for school-aged children and adolescents [113]. However, side effects such as decreased appetite, weight loss, and insomnia are common, and careful monitoring is necessary to mitigate these risks [115]. There continues to be concern about long-term use and the potential for misuse, though research does not indicate a strong link between stimulant medication use and future substance abuse [113].

For individuals who do not respond well to stimulants, non-stimulant medications provide an alternative. Atomoxetine, a selective norepinephrine reuptake inhibitor, has been found to reduce symptoms, though it typically takes longer to achieve noticeable effects compared to stimulants [112]. Other non-stimulant options, such as guanfacine and clonidine, are sometimes prescribed, particularly for children with co-occurring conditions like anxiety or sleep disturbances [115]. While these medications are generally well-tolerated, they may cause side effects such as drowsiness, fatigue, and low blood pressure [113]. Research indicates that non-stimulants are less effective than stimulant medications but can be useful for specific cases where stimulants are not suitable [112].

While medication plays a central role in symptom management, combining pharmacological treatment with behavioral therapy leads to better long-term outcomes. Studies have shown that behavioral interventions, including cognitive-behavioral therapy, skills training, and contingency management, improve academic performance and social functioning [114]. Behavioral therapy appears to have a greater impact on overall impairment, while medication is more effective in reducing core ADHD symptoms [113]. Adolescents, in particular, benefit from a multimodal approach that incorporates both medication and therapy, as they face additional challenges related to autonomy, academic demands, and social relationships [112].

The long-term use of ADHD medications continues to be an area of ongoing research. While stimulants provide rapid symptom relief, potential concerns include growth suppression, cardiovascular effects, and adherence issues [115]. Many adolescents discontinue treatment despite ongoing symptoms, which can negatively impact academic and social development [113]. Studies emphasize the importance of regular monitoring and individualized treatment adjustments to ensure optimal management and minimize risks [112]. The need for additional research on long-term neurodevelopmental outcomes remains critical, as current studies focus primarily on short-term efficacy [114].

Pharmacological treatments for ADHD remain the most effective approach for symptom management, with stimulant medications being the first-line option. Non-stimulant medications serve as viable alternatives for individuals with contraindications or poor stimulant tolerance. The combination of medication and behavioral therapy provides the most comprehensive treatment approach, addressing both core symptoms and broader functional impairments. Despite the effectiveness of these treatments, long-term studies are necessary to assess their impact on neurodevelopment and overall well-being.

### Medical Treatment of Anxiety and Depression in Youth

Depression and anxiety are among the most prevalent psychiatric disorders in children and adolescents, often resulting in significant impairment in daily functioning, academic performance, and social interactions. Effective treatment typically involves a combination of pharmacological and non-pharmacological approaches, with selective serotonin reuptake inhibitors (SSRIs) and cognitive-behavioral therapy (CBT) being the most widely supported interventions.

Pharmacotherapy plays a critical role in the treatment of moderate to severe depression in youth. Selective serotonin reuptake inhibitors (SSRIs) are the first-line pharmacological treatment, with fluoxetine (Prozac) and escitalopram (Lexapro) being the only antidepressants approved by the U.S. Food and Drug Administration (FDA) for use in children and adolescents [116]. Fluoxetine is approved for patients as young as eight years old, while escitalopram is approved for those aged twelve and older. SSRIs work by increasing serotonin levels in the brain, which helps regulate mood, emotions, and anxiety.

While SSRIs are generally well tolerated, they do carry potential side effects, including nausea, headaches, weight changes, and an increased risk of suicidal thoughts, particularly in the early stages of treatment. As a result, careful monitoring for suicidality is essential, especially during the first few weeks of treatment [116]. Studies indicate that combining SSRIs with cognitive-behavioral therapy (CBT) yields better outcomes than medication alone, particularly in reducing symptom severity and preventing relapse (Strawn et al.) A growing body of research supports the combination of SSRIs and CBT as the most effective treatment for both depression and anxiety in youth [117]. The benefits of CBT become more pronounced over time, with studies showing significant symptom reduction by the 12-week mark of treatment [117]. However, individual responses to treatment vary, and clinicians must tailor interventions based on patient characteristics such as severity of symptoms, comorbidities, and previous treatment history.

Anxiety disorders in children and adolescents are commonly treated with SSRIs, which are both safe and effective for generalized anxiety disorder (GAD), social anxiety disorder, and separation anxiety disorder [118]. Sertraline (Zoloft), fluoxetine (Prozac), and fluvoxamine (Luvox) are among the most frequently prescribed SSRIs for pediatric anxiety disorders. Serotonin-norepinephrine reuptake inhibitors (SNRIs), such as venlafaxine (Effexor XR) and duloxetine (Cymbalta), also have some empirical support as alternative pharmacological options.

Long-term use of SSRIs remains a subject of ongoing research, particularly regarding their effects on neurodevelopment. While SSRIs are generally considered safe, some concerns exist regarding their impact on emotional processing and behavior over extended periods [118]. Physicians should regularly assess treatment efficacy and adjust dosages or switch medications if necessary.

### Medical Management of Body Dysmorphic Disorder

BDD is a severe psychiatric condition that often emerges during adolescence and significantly impairs social, academic, and emotional functioning. It is characterized by a persistent preoccupation with perceived flaws in physical appearance that are typically unnoticed by others. BDD leads to compulsive behaviors such as mirror checking, excessive grooming, and camouflaging [119]. The disorder is frequently underdiagnosed due to limited clinical awareness and stigma, and untreated cases often follow a chronic trajectory, increasing the risk of severe distress, functional impairment, and suicidality [120]. Given these concerns, early intervention with evidence-based treatments, primarily cognitive behavioral therapy (CBT) and selective serotonin reuptake inhibitors (SSRIs), is critical.

CBT is the gold-standard psychotherapy for BDD in children and adolescents, with extensive evidence supporting its efficacy. CBT interventions for BDD focus on restructuring maladaptive thought patterns, reducing compulsive behaviors, and addressing avoidance tendencies. Specific techniques, such as exposure and response prevention (ERP), mirror retraining, and cognitive restructuring, are highly effective in modifying the negative self-perceptions that drive BDD symptoms [121]. Studies conducted at specialist clinics in Stockholm and London demonstrated that CBT leads to significant reductions in BDD severity among adolescents, with improvements persisting up to one year after treatment [122]. Additionally, therapist-guided internet-based CBT is effective in cases where access to in-person treatment is limited, with benefits comparable to traditional face-to-face therapy [123].

Pharmacotherapy is another key component of BDD treatment, particularly for individuals with severe or treatment-resistant symptoms. SSRIs, including fluoxetine, sertraline, and escitalopram, have been demonstrated to significantly reduce BDD-related obsessive thoughts and compulsive behaviors [124]. These medications are typically prescribed at higher doses than those used for depression, and treatment duration must be extended for optimal results. Clinical trials have shown response rates to SSRIs ranging from 53% to 65%, with greater efficacy observed when combined with CBT [125]. Additionally, for individuals who do not respond adequately to SSRIs alone, augmentation strategies, such as the addition of atypical antipsychotics like aripiprazole, have been explored, though more research is needed to establish their efficacy [119].

Comorbidities are highly prevalent among individuals with BDD and must be considered when developing treatment plans. Adolescents with BDD frequently experience co-occurring conditions such as MDD, OCD, anxiety disorders, and eating disorders [120]. Additionally, research suggests a notable overlap between BDD and neurodevelopmental disorders such as ASD and ADHD, with one study reporting ASD comorbidity rates as high as 16% among young people with BDD [119]. These findings highlight the necessity of comprehensive assessments and personalized treatment approaches to address the full spectrum of psychiatric symptoms present in affected individuals.

Despite the availability of effective treatments, many adolescents with BDD do not receive appropriate care. The disorder is often mistaken for typical adolescent body image concerns, leading to delays in diagnosis and treatment [120]. Furthermore, individuals with BDD are more likely to seek cosmetic or dermatological procedures rather than psychiatric intervention, which can exacerbate symptoms and increase distress when the perceived flaws remain uncorrected [121]. The chronic nature of BDD, coupled with its association with high rates of suicidality, underlines the urgency of improving early detection and expanding access to specialized mental health services.

## Conclusions

Social media and technological advancements’ impact on adolescent mental health is complex. It can be both a risk factor and a valuable support system. Excessive and problematic use has been linked to increased rates of MDD, anxiety, and mood dysregulation, while also exacerbating symptoms of ADHD, bipolar disorder, and BDD. Simultaneously, digital platforms provide opportunities for social connection, peer support, and mental health management, particularly for individuals with ASD and those seeking online mental health communities. The challenge is finding a balance. Although social media offers benefits, it also poses risks like addiction, negative social comparison, cyberbullying, and impulsive online behaviors [125].

A multidisciplinary approach is essential in addressing these challenges. In conjunction with one another, policymakers, educators, researchers, and technology companies could, by pooling their expertise and resources, develop digital literacy programs, mental health education initiatives, and evidence-based interventions to promote responsible social media use. Digital platforms should integrate content moderation systems, AI-driven safety measures, and mental health awareness campaigns to reduce harm while fostering a healthier online environment. Alongside these structural interventions, adolescents must be equipped with the skills to self-regulate their digital habits with the help of parental guidance and peer-led advocacy efforts [126].

Future research should prioritize longitudinal studies to better understand the long-term psychological effects of social media use and identify the factors differentiating adaptive from maladaptive digital behaviors. In parallel, greater attention should be given to technology-based interventions, such as mood-tracking applications for bipolar disorder or digital tools designed to curb compulsive social media use, which holds promise for more tailored and scalable mental health support. As digital platforms continue to evolve, our understanding of their psychological impact must evolve with them. Developing evidence-informed policies, educational initiatives, and accessible support systems will be essential in creating online environments that foster mental well-being in adolescents [127]. Ultimately, protecting youth mental health in the digital age will require innovation and a cultural shift toward more mindful and responsible engagement with technology. Protecting the mental health of today’s youth is not just a medical or technological concern; it is a societal imperative that demands urgent, coordinated action across every level of influence. As we navigate the ever-evolving digital frontier, we must ensure that young minds are connected and protected.

## Figures and Tables

**Figure 1:**
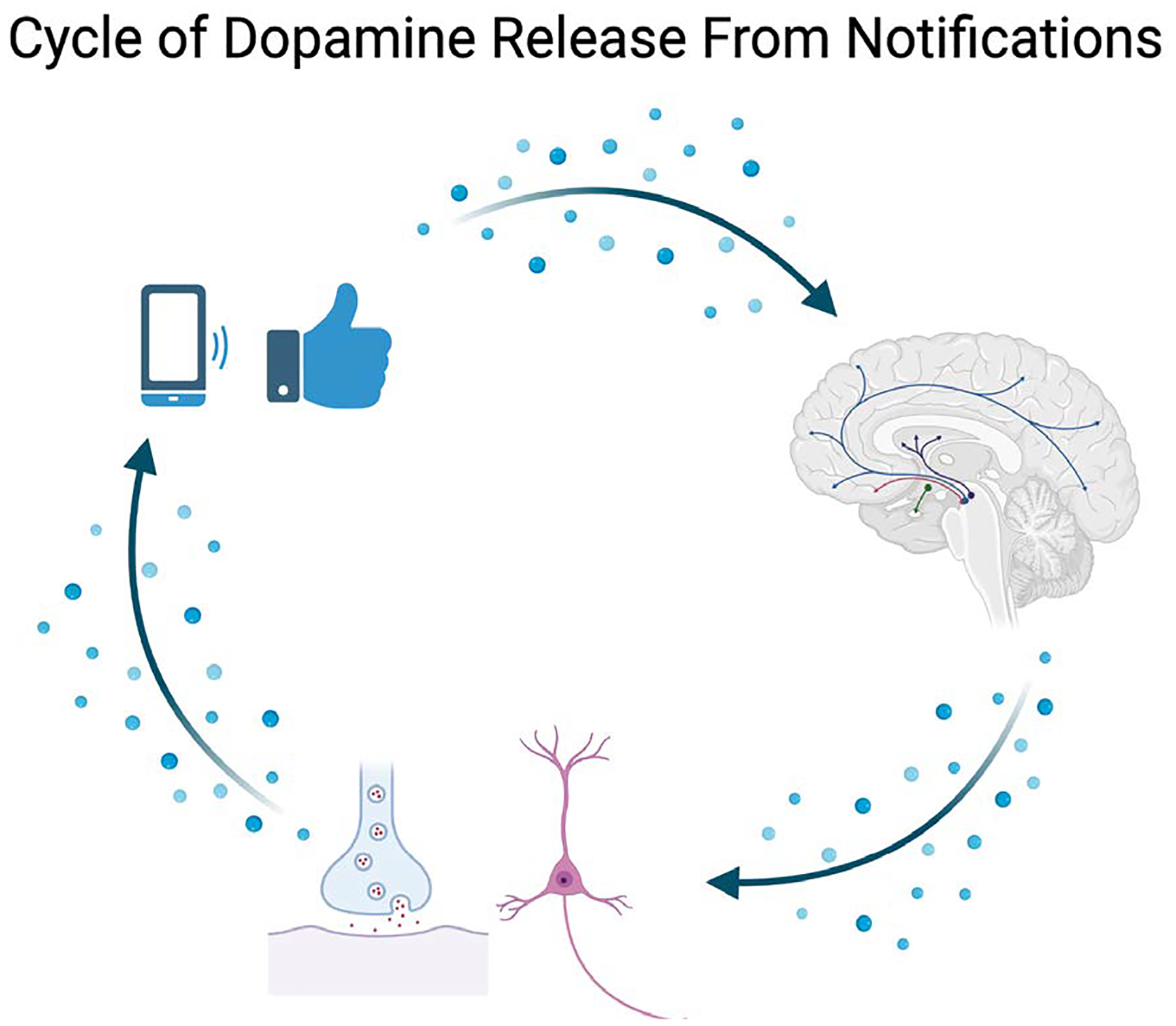
Dopamine release in the mesolimbic pathway promoting an addictive cycle of the social media notification reward system.

**Figure 2:**
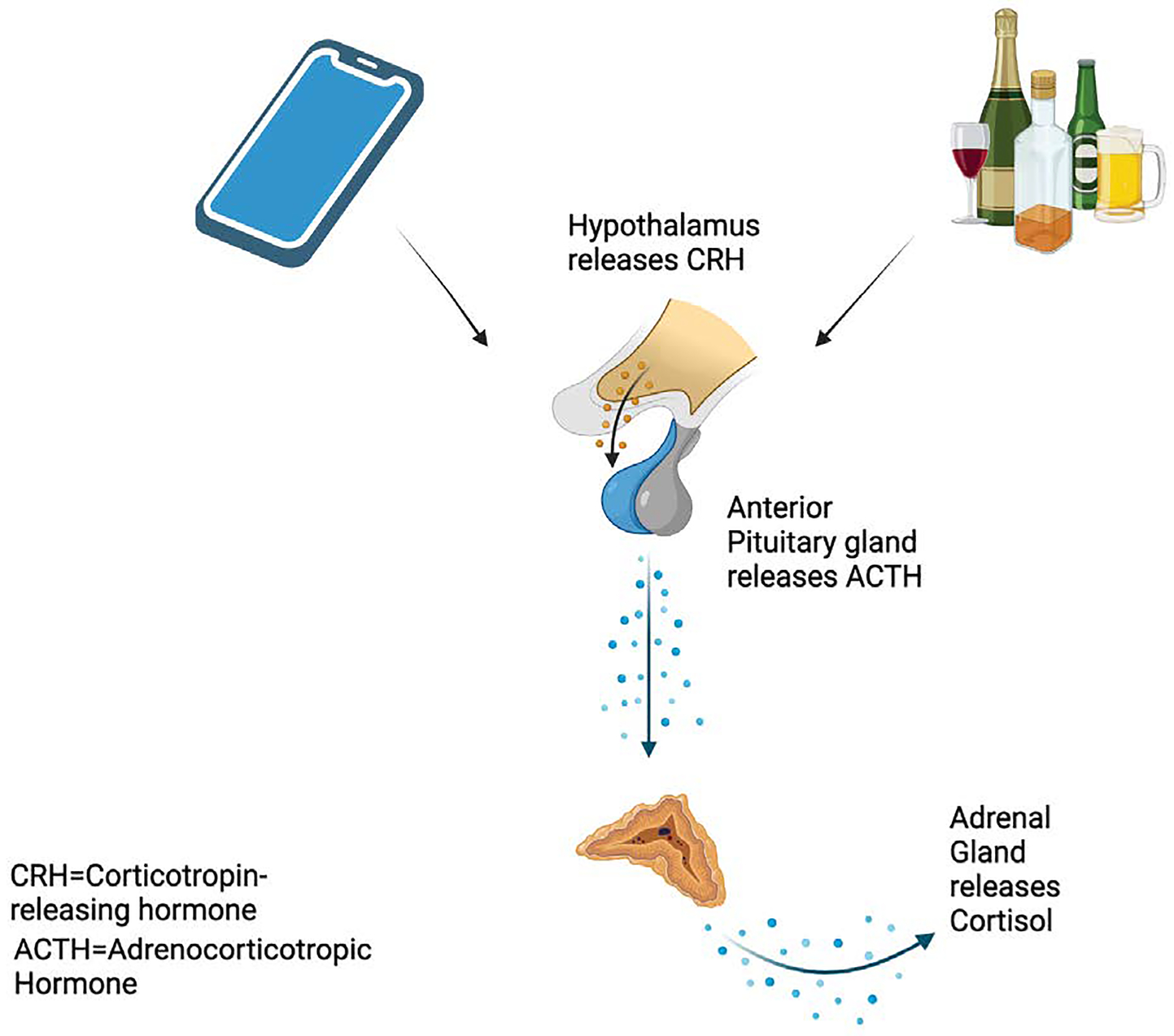
Hypothalamic-pituitary access chronically activated in addictions such as social media use or alcohol use.

**Figure 3:**
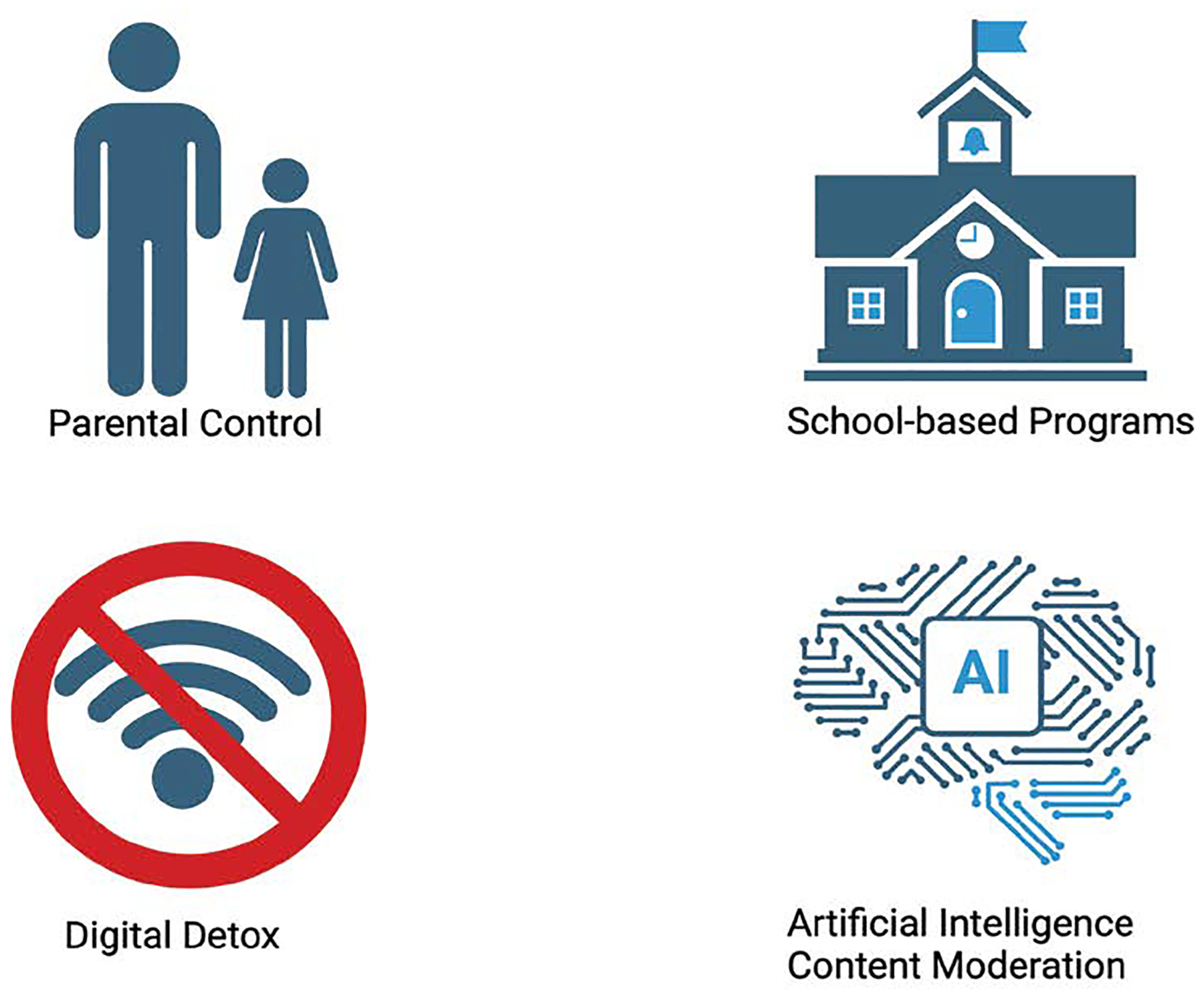
Strategies to mitigate the harmful effects of excessive screen time in the youth including AI, digital detox programs, parental restrictions/control, and institutionalized education on healthy habits.

## Key Points

- Excessive and unrestricted social media use is linked to increased occurrence of several many mental health disorders.
- The mesolimbic pathway and dopamine reward system is linked to the addiction of social media.
- ADHD symptoms and impulsive behaviors are exacerbated by excessive digital media use in adolescents.
- Youth exposure to content condoning substance abuse is more likely to engage in risky behavior including drug and alcohol use
- Online anonymity has fostered an environment that allows for increased cyberbullying, linked to higher rates of depression, anxiety, and negative emotions
- Digital detox, screen time limitations, and parental control could help limit the negative effects of technology in youth.
- CBT and medications are both effective treatments for youth mental health disorders, and even more effective when used in combination.
- Technology and AI based programs are also useful resources in moderating content in digital environments.
